# Cognitive Impairment in ANCA-Associated Vasculitis: A Cross-Sectional Pilot Study

**DOI:** 10.3390/jcm14103582

**Published:** 2025-05-20

**Authors:** Marion Camard, Ana Moises, Katia Bourdic, Laura Venditti, Christian Denier, Julien Henry, Raluca Sterpu, Perla David, Mathilde De Menthon, Olivier Lambotte, Anne-Cécile Petit, Matthias Babin, Nicolas Noel, Fanny Urbain

**Affiliations:** 1 Groupe Hospitalier Universitaire Paris Saclay, Internal Medecine and Clinical Immunology Department, AP-HP, Hôpital Bicêtre, F-94275 Le Kremlin-Bicêtre, France; marion.camard@aphp.fr (M.C.); fanny.urbain@aphp.fr (F.U.); 2 Groupe Hospitalier Universitaire Paris Saclay, Neurology Department, AP-HP, Hôpital Bicêtre, F-94275 Le Kremlin-Bicêtre, France; 3 Groupe Hospitalier Universitaire Paris Saclay, Rheumatology Department, AP-HP, Hôpital Bicêtre, F-94275 Le Kremlin-Bicêtre, France; 4 Groupe Hospitalier Universitaire Paris Saclay, Internal Medecine and Clinical Immunology Department, AP-HP, Hôpital Antoine-Béclère, F-92140 Clamart, France; 5 Inserm, CEA, Center for Immunology of Viral, Auto-immune, Hematological, Bacterial Diseases (IMVA-HB/IDMIT/UMRS1184), Université Paris Saclay, F-94275 Le Kremlin-Bicêtre, France; 6 Groupe Hospitalier Universitaire Paris Psychiatrie et Neurosciences, Pôle Hospitalo-Universitaire Psychiatrie Paris 15, Hôpital Sainte-Anne, F-75014 Paris, France; 7 School of Medicine, Paris Cité University, F-75006 Paris, France; 8 Pasteur Institute, CNRS UMR 3571, Perception and Action Unit, Université Paris Cité, F-75015 Paris, France; 9 Groupe Hospitalier Universitaire Paris Saclay, Neuroradiology Department, AP-HP, Hôpital Bicêtre, F-94275 Le Kremlin-Bicêtre, France

**Keywords:** cognitive dysfunction, mild cognitive impairment, neuropsychological tests, antineutrophil cytoplasmic antibody-associated vasculitis

## Abstract

**Objectives:** Antineutrophil cytoplasmic antibody (ANCA)-associated vasculitis (AAV) comprises rare systemic vasculitides that can present with cognitive dysfunction. However, data on the screening and characterization of cognitive dysfunction in AAV remain limited. **Methods:** Cognitive complaints in AAV patients were screened using self-report questionnaires. Objective cognitive impairment was assessed with a standardized neurocognitive test battery. Results were compared with clinical evaluations, brain MRI findings, treatment history, and neuropsychiatric symptoms. All test results were standardized for the overall population. **Results:** Twelve patients (five women, seven men) with a median [IQR] age of 68 [59–71] and a median [IQR] disease duration of 92 months [55–127] were included. None of the patients showed evidence of vasculitis activity on brain MRI. Cognition was assessed using a standardized neurocognitive test battery in all patients except one. Four patients (36%) were found to have cognitive impairment, defined as three or more altered tests. The most affected functions were attentional and executive, with the d2-R (4/4), Rey–Osterrieth Complex Figure Delayed Recall (3/4), and Trail Making Test Part B (3/4) showing the most frequent deficiencies. Objective cognitive disorders were not associated with self-reported cognitive complaints. No significant association was found between cognitive impairment and vasculitis activity or sequelae, corticosteroid and immunosuppressive treatments, or neuropsychiatric symptoms. **Conclusions:** This study highlights the presence of cognitive impairments in AAV, predominantly affecting attentional and executive functions, which may reflect vascular involvement. Early and tailored approaches to cognitive screening and management are essential to improve patient care and quality of life.

## 1. Introduction

Antineutrophil cytoplasmic antibody (ANCA)-associated vasculitis (AAV) is a group of rare autoimmune diseases affecting small and medium-sized vessels [1]. AAV can involve the central nervous system (CNS), either as a direct manifestation of systemic vasculitis or through secondary mechanisms such as inflammation and vascular damage in other organs [2]. CNS involvement in AAV is heterogeneous and challenging to diagnose, with manifestations including intraparenchymal or meningeal hemorrhage, stroke, or pachymeningitis. Among these, cognitive impairment has been increasingly reported, but remains poorly understood and insufficiently characterized [2,3].

Cognitive impairment in AAV has been described in a few studies, including cases without visible lesions, suggesting a potential role for systemic inflammation in neurocognitive dysfunction [2,3]. This aligns with findings in other systemic inflammatory diseases, such as lupus and rheumatoid arthritis, where elevated levels of proinflammatory cytokines have been associated with cognitive decline [4,5].

Despite its significant impact on quality of life, cognitive dysfunction remains underdiagnosed in AAV due to a lack of appropriate screening tools and patient systematic complaints. Neuropsychiatric symptoms or disorders, such as depression, anxiety, fatigue, or pain, frequently reported in AAV, further affect patients’ quality of life [6].

This study aimed to identify and characterize cognitive disorders in AAV patients followed in a tertiary university hospital, and to explore their association with disease activity, sequelae, cognitive complaints, and neuropsychiatric symptoms.

## 2. Materials and Methods

### 2.1. Patient Enrollment and Diagnostic Criteria

Patients over 18 years old with AAV, diagnosed according to the ACR 2022/EULAR classification criteria, were prospectively enrolled between January 2023 and June 2024 from two hospitals within the Paris-Saclay Hospital Group [7,8,9]. Eligibility required cognitive complaints or suspected cognitive impairment by the referring physician. Exclusion criteria included a history of cognitive impairment (abnormal cognitive assessment in medical records), limited knowledge of French, and legal protection. Neurocognitive assessments were performed during the annual vasculitis evaluation, which included clinical examination, blood tests, brain MRI, and assessments of AAV activity (BVAS—Birmingham Vasculitis Activity Score Version 3) and sequelae (VDI—Vasculitis Damage Index). Additional evaluations included inflammatory markers (C-reactive protein), ANCA levels, and cumulative corticosteroid doses, collected from medical records. Patients also completed the three-questions subjective cognitive complaint (Q3PC), adapted from the European EACS Guidelines 12.0, which is a short screening questionnaire assessing complaints of memory loss, difficulty concentrating, and attentional disorders [10].

### 2.2. Ethics

This study was conducted according to the guidelines of the Declaration of Helsinki, and was registered with CNIL approval (National Commission on Informatics and Liberties): registration no. 2226405 v 0. The study was approved by the Comité de Protection des Personnes (CPP) IDF1 (protocol code CPPIDF1-2022-DI65- IC-Cas 3, 9 August 2022). All patients provided written informed consent to participate.

### 2.3. Cognitive Evaluation

Neurocognitive assessments were standardized and conducted by a specialized neuropsychologist (AM), in 2 h sessions. Patients completed the FACT-Cog (Functional Assessment of Cancer Therapy–Cognitive Function), a self-administered oncology questionnaire assessing perceived cognitive changes [11]. Memory complaints were assessed using the 39-item French MacNair hetero-assessment scale, with significant complaints defined as a score > 54 [12]. A semi-structured interview collected information on educational, occupational, and family background, and functional symptoms.

Neuropsychological assessment was conducted using a standardized battery of tests, selected based on the recommendations of the French group for general cognitive assessment [13]. The neurocognitive test battery included the Montreal Cognitive Assessment (MoCA), the Rey–Osterrieth Complex Figure (ROCF), the Free and Cued Selective Reminding Test (FCSR), the Digit Span test (WAIS—Wechsler Adult Intelligence Scale IV), the Trail Making Test (TMT), the Digit–Symbol-Coding test (WAIS III), the d2-R test, the Stroop test, and semantic and phonemic verbal fluency tests (Supplementary Table S1). A MoCA score below 26/30 was considered abnormal. Each test was adjusted for age, biological sex, and education level according to published norms. Cognitive impairment was defined as insufficient performance on three or more tests (below the fifth percentile or 1.65 standard deviations), excluding the MoCA, used only as a screening tool. Intermediate results were for scores between the 5th and 10th percentiles. Results were reviewed with patients, and follow-up options, including psychological support, metacognitive work, and compensatory strategies, were offered.

### 2.4. Neuropsychiatric Evaluation

Neuropsychiatric symptoms, which may affect cognitive function, were assessed using hetero-assessment questionnaires: the Hospital Anxiety and Depression Scale (HADS) for anxiety and depression (significant symptoms: >8; mild: 8–10; moderate: 11–14; severe: 15–21), the Insomnia Severity Index for insomnia (no insomnia: 0–7; subthreshold: 8–14; moderate: 15–21; severe: 22–28), and the Visual Analog Scale for pain (moderate: 4–5; severe: >6).

### 2.5. Brain MRI

Brain MRI, including diffusion, 3D FLAIR, 3D TOF, SWI, and T1 FAT-SAT (with/without gadolinium), was evaluated by an experienced neuroradiologist (MB) for vascular sequelae and atrophy.

### 2.6. Statistical Analysis

Continuous variables were reported as medians [IQR] and categorical variables as frequencies (%). Between-group comparisons were performed using Mann–Whitney U and Fisher’s exact tests. Correlations were assessed using Spearman’s rank correlation coefficients. Statistical analyses were performed using R (version 4.3.3), with *p* < 0.05 considered significant.

## 3. Results

We included 12 patients (5 women and 7 men) with a median age of 68 [59–71] years. The median time since diagnosis was 92 [55–127] months. Clinical, biological, disease activity at the time of diagnosis (as assessed by the BVAS score), and brain MRI characteristics are summarized in Table 1. Most patients had inactive AAV at enrollment (BVAS score of 0); two patients had low disease activity (BVAS scores of 1 and 5). Brain MRI showed no signs of active CNS vasculitis.

Severe depression (HADS-D: 20/21) prevented 1 patient from completing the neuropsychological assessment, reducing the study population to 11. Subjective self-reported cognitive complaints were reported by 6/11 (55%) patients using Q3PC and 4/11 (50%) using FACT-Cog, with concordance in 7/11 cases (64%). Memory complaints on the MacNair hetero-assessment scale were reported by 3/11 patients (27%), with no correlation with Q3PC or FACT-Cog.

**Figure 1:**
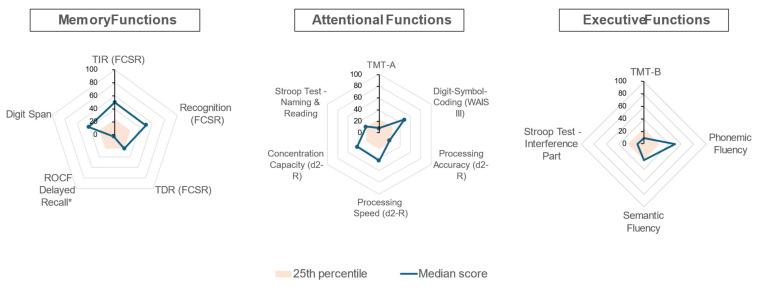
Median scores of all patients across the cognitive domains tested, expressed in percentiles. Cognitive test scores were adjusted for age, sex, and educational norms and compiled into three radar charts, each representing a primary cognitive domain: memory functions, attention functions, and executive functions. The closer the median score is to the center, the greater the median impairment in that cognitive domain. *: Four out of seven patients with deficient ROCF Delayed Recall also demonstrated inadequate or intermediate performance on the ROCF copying task, suggesting planning deficits rather than encoding deficits related to incidental learning. Abbreviations: FCSR: Free and Cued Selective Reminding Test; TDR: Total Delayed Recall (part of the Free and Cued Selective Reminding Test); TIR: Total Immediate Recall (part of the Free and Cued Selective Reminding Test); TMT: Trail Making Test; WAIS: Wechsler Adult Intelligence Scale.

Screening for mild cognitive impairment with the MoCA revealed a median score of 27/30 [23.5–27.5], with 4/11 patients (36%) scoring below the threshold of 26/30. Figure 1 illustrates the median scores for all patients by category (memory, attention, executive function), expressed in percentiles and normalized for age, biological sex, and education level. Deficits were more pronounced in attentional and executive functions. Notably, seven patients (64%) failed the ROCF Delayed Recall, although four of these had previously demonstrated inadequate or intermediate performance on the ROCF copying task, likely due to encoding and planning deficits. The d2-R test identified the most severe attentional impairments, while executive function deficits were observed in the TMT-B (4/11; 36%) and the Stroop interference task (2/11; 14%) (Supplementary Table S2). Neurocognitive test results were not influenced by cognitive complaints (Supplementary Table S3).

Finally, 4/11 patients (36%) met the cognitive impairment criteria (≥3 insufficient tests, excluding MoCA), including 1 with a normal MoCA score. The most frequent deficits were in the d2-R (4/4), ROCF Delayed Recall (3/4), and TMT-B (3/4). Three patients (75%) reported cognitive complaints (FACT-Cog or Q3PC), while one was anosognosic. One patient had Scheltens grade 3 hippocampal atrophy, with predominant memory impairment without neuropsychiatric symptoms. No significant differences in cognitive impairment were observed based on sex, age, disease duration, current or cumulative corticosteroid use, immunosuppressive treatments, or VDI score (see Supplementary Table S4). Likewise, common comorbidities such as diabetes or hypertension did not appear to influence cognitive performance.

We examined the relationship between neuropsychiatric symptoms and cognitive impairment (Supplementary Table S5). Severe depression was observed in 2/11 patients (18%), and mild-to-severe anxiety in 7/11 (64%). Among patients with cognitive impairment, one reported severe anxiety, one severe depression and insomnia, one moderate insomnia, and one no symptoms. Objective cognitive impairment was not significantly associated with anxiety, depression, insomnia, or pain. Cognitive complaints, as assessed by the FACT-Cog and MacNair scales, were significantly correlated with insomnia, with Spearman correlation coefficients of r = 0.66 (*p* = 0.01) and r = 0.73 (*p* = 0.002), respectively.

## 4. Discussion

This study presents the first cross-sectional evaluation of cognitive impairment in AAV patients in France. Despite stringent criteria (the presence of at least three insufficient test scores), 4/11 (36%) patients were found to have cognitive impairment. Data on the prevalence of cognitive disorders in AAV are limited, with two studies using neurocognitive battery tests estimating it at 30% (13/43 and 21/60) [2,3]. Both identified similar impairments in executive functions, abstract reasoning, and attention, consistent with our findings.

Cognitive impairment was identified despite stable cerebral MRI and inactive vasculitis. No association was found with neurological sequelae assessed by the VDI score, which poorly reflects cognitive impairment. A previous monocentric study using the MoCA reported decreased scores in 60% of inactive AAV patients, correlated with higher VDI scores. In contrast, we found no such link, likely due to our smaller cohort and fewer sequelae (median VDI score: 2) [14]. This finding highlights the importance of systematic cognitive screening in AAV patients, even without apparent neurological sequelae.

We observed notable impairments in attention and executive function (Figure 1), with test variability reflecting the complexity of assessing these domains. Phonemic and semantic fluency tests, which assess spontaneous flexibility, were relatively preserved, while the TMT Part B and Stroop test highlighted deficits in reactive flexibility and response inhibition, respectively. This executive and attentional impairment appears more consistent with a vascular origin, supported by cognitive patterns, relatively preserved memory, and normal brain MRI [15]. Similar profiles have been described in other chronic inflammatory or vascular diseases, such as chronic kidney disease and rheumatoid arthritis [3]. Effective therapies for AAV have significantly reduced mortality and transformed these diseases into chronic conditions characterized by organ damage, residual inflammation, prolonged immunosuppressive treatment, and risk of relapse—all potential contributors to cognitive dysfunction [2,16]. Our findings reinforce the vascular nature of AAV and its link to cognitive impairment.

Interestingly, one patient with cognitive impairment was anosognosic (lacking cognitive complaints), exhibited no neuropsychiatric symptoms, and had a normal brain MRI. To assess self-reported complaints, we adapted the Q3PC (used in HIV and COVID-19) and the FACT-Cog (commonly used in oncology), since no such tools exist for AAV. However, both showed inconsistent results with objective neurocognitive tests in AAV patients [10,11]. The MacNair hetero-assessment scale, focused on memory complaints, showed no correlation with other assessments. These findings highlight the need for systematic cognitive assessments, especially for anosognosic patients, and for tools targeting attentional and executive disorders. Cognitive complaints might indirectly reflect neuropsychiatric symptoms, as evidenced with their correlation with insomnia.

Neuropsychiatric symptoms were prevalent in our cohort, particularly anxiety (Supplementary Table S5). A cross-sectional study of 60 AAV patients similarly identified anxiety as the most common neuropsychiatric comorbidity, with depression also being prevalent [3]. A meta-analysis estimated a pooled prevalence of depression in small vessel vasculitis at 28%, with significant variability across studies [17]. Inflammatory cytokines may disrupt neuronal pathways, leading to depression by altering the hypothalamic–pituitary–adrenal axis and neurotransmitter balance [18]. Cytokine levels could not be assessed in our study. However, the possibility of persistent low-grade inflammation in patients with AAV, even during remission, highlights the need for a more detailed characterization of inflammatory parameters beyond CRP, the only marker available in our study. These imbalances may contribute to cognitive and neuropsychiatric disorders, as described, for example, in COVID-19 [19].

In our study, 7/11 (64%) of patients reported insomnia. Although fatigue was not directly assessed, it is influenced by pain, inflammation, and insomnia, which were measured [6]. A functional brain MRI study suggests that fatigue in AAV may involve the striato–thalamo–frontal brain structures [20]. Additionally, a 2021 study demonstrated the feasibility and acceptability of a physical activity intervention for fatigue management in AAV patients [21]. Treatable neuropsychiatric symptoms affect cognitive functions, highlighting the need for screening and management to improve quality of life. The recent AAV-PRO patient-reported score evaluates health-related quality of life and screens for cognitive disorders, anxiety, fatigue, and depression, supporting comprehensive care in AAV [22].

The limitations of our study include a small cohort size, which reflects the low prevalence of AAV. Additionally, the absence of a control group may be questioned. However, this limitation was mitigated by the use of a standardized neurocognitive test battery, normed on healthy French populations, with scores adjusted for age, sex, and educational level, as described in reference [13]. This battery is endorsed by French expert recommendations for the evaluation of cognitive impairment in vascular diseases, and its normative data allow for individualized interpretation. Functional MRI was unavailable and inflammation markers were limited to C-reactive protein, preventing exploration of local inflammation through cerebrospinal fluid analysis of neurotransmitters, blood–brain barrier integrity, or proinflammatory cytokines. Lastly, the length of the neurocognitive test battery may influence the results due to patient fatigue, particularly in a population already prone to fatigue and neuropsychiatric symptoms.

## 5. Conclusions

In conclusion, this pilot study identified a notable prevalence of cognitive impairment in AAV patients, primarily affecting attention and executive functions, with mechanisms yet to be determined. Early detection of cognitive disorders should be a priority, along with management of neuropsychiatric comorbidities as part of comprehensive chronic disease care.

## Figures and Tables

**Table 1:** Patients’ characteristics.

		All Patients n = 12
Female sex, n (%)		5 (42)
Age (years) [IQR]		68 [59–71]
Body Mass Index, (kg/m^2^) [IQR]		24.75 [4–39]
Substance use	Active smoking	0 (0)
	Alcohol	0 (0)
Type of AAV vasculitis, n (%)		
	GPA	8 (67)
	MPA	1 (8)
	EGPA	3 (25)
ANCA positivity, n (%)		
	Anti-PR3	5 (42)
	Anti-MPO	6 (50)
	Non-specific	1 (8)
Organ involvement at diagnosis, n (%)		
	Peripheral nervous system	7 (58)
	Renal failure with proteinuria	4 (33)
	Pulmonary hemorrhage	2 (17)
BVAS score at diagnosis, median [IQR]		10 [10–18.5]
Vasculitis relapse, n (%)		7 (58)
Treatments		
Induction treatment receiveda, n (%)		
	Plasma exchanges	1 (8)
	Cyclophosphamide	6 (50)
	Rituximab	4 (33)
Received cumulative corticosteroid dose, grams, median [IQR]		22.3 [15.7–26.6]
		92 [55–127]
Current status	Corticosteroids	5 (42)
Post-diagnosis delay, (months) [IQR]	Immunosuppressive treatments	4 (33)
Current treatments, n (%)	Psychotropic treatments	5 (42)
		
	Sequelae of neuropathy	7 (58)
		
Vasculitis activity	0	10 (83)
	≥1	2 (17)
		2 [1–2.5]
BVAS score, n (%)		1 (8)
		0 (0)
VDI score [IQR]		
ANCA positivity, n (%)	No sign of vasculitis activity	12 (100)
CRP > 5 mg/L, n (%)	Fazekas grade 2 vascular leukoencephalopathy	2 (17)
Brain MRI results, n (%)	Hippocampal atrophy (Scheltens grade 2 or 3)	3 (25)
	Vascular sequelae (deep lacunes and junctional sequelae)	0 (0)
	Microbleeds	3 (25)
	Cortico-subcortical sequelae	0 (0)

[IQR]: Median with interquartile range. a: Includes initial diagnosis and relapses, AAV: ANCA-associated vasculitis, BVAS: Birmingham Vasculitis Activity Score, CRP: C-reactive protein, VDI: Vasculitis Damage Index. a: Includes initial diagnosis and relapses.

## Data Availability

The data underlying this article will be shared on reasonable request to the corresponding author.

## Supplementary Materials

The following supporting information can be downloaded at https://www.mdpi.com/article/10.3390/jcm14103582/s1: Table S1: Summary of neurocognitive test battery; Table S2: Cognitive evaluation score. Table S3: Self-Report questionnaire and cognitive trouble association. Table S4: Factors associated with cognitive impairment. Table S5: Psychopathological symptoms associated with cognitive impairment.
