# Supplementary material for: Cognitive Impairment in ANCA-Associated Vasculitis: A Cross-Sectional Pilot Study
Source: J Clin Med. 2025 May 20;14(10):3582. doi: 10.3390/jcm14103582 (PMC12112563; doi:10.3390/jcm14103582)
Supplement: Supplementary file 1 [file jcm-14-03582-s001.zip › jcm-3625726-supplementary.pdf]

Supplementary Table S1: Summary of neurocognitive test battery

Cognitive Domain	Test Name	Test Evaluation	Test Procedure
**Global Functioning**	MoCA	Rapid and global cognitive assessment	Patient responds to instructions that evaluate several subcategories including attention, memory, language, visuoconstructive ability, abstraction, calculation, and orientation.
**Instrumental Functions**	ROCF copying task	Visuoconstructive and visuospatial skills, attention	Patient copies a figure and then reproduces it from memory immediately.
**Memory Functions**	FCSR *(French version)*	Verbal episodic memory	Patient memorizes a list of 16 words, each associated with a semantic category, followed by immediate cued recall, three trials of free and cued recall, delayed recall after 20 minutes, and a recognition phase.
	ROCF Delayed Recall	Visual episodic memory	Patient reproduces the figure from memory after a 3-minute delay.
	Digit Span (WAIS IV)	Short-term and working memory	Patient repeats series of numbers in both forward and backward sequences.
**Attentional Functions**	TMT-A	Visual attention speed	Part A: Patient connects numbered dots.
	Digit–Symbol-Coding (WAIS III)	Evaluation of processing speed and visuomotor skills	Patient matches symbols with numbers within a time limit.
	d2-R test	Attention and concentration	Patient crosses out specific targets among distractors. This assesses concentration capacity (overall performance considering speed and errors), processing speed (working speed during the test), and processing accuracy (percentage of errors relative to targets processed).
	Stroop test—Part color naming and reading	Attentional control	Patient reads as many words as possible and names the color of as many rectangles as possible.
**Executive Functions**	TMT-B	Processing speed and cognitive flexibility	Part B: Patient alternates between numbers and letters.
	Phonemic and semantic fluency	Organization and retrieval strategy	Patient names as many words as possible within a given time that belong to a semantic category or start with a specific letter.
	Stroop test	Cognitive inhibition	Patient names the color of the ink used to print the words, while the word itself is a different color.

Abbreviations: FCSR: Free and Cued Selective Reminding Test; MoCA: Montreal Cognitive Assessment; ROCF: Rey–Osterrieth Complex Figure; TMT: Trail Making Test; WAIS: Wechsler Adult Intelligence Scale

Supplementary Table S2: Cognitive evaluation score

Cognitive Domain	Cognitive Tests [a]	AAV *n = 11*
Global Functioning	**MoCA**	
	< abnormal 26/30	4 (36)
	> normal 27/30	7 (64)
Instrumental Functions	**ROCF copying task**, *n (%)*	
	Satisfying	6 (55)
	Intermediate	3 (27)
	Insufficient	2 (18)
Memory Functions	**FCSR (French version),** *n (%)*	
	Total Immediate Recall insufficiency	1 (9)
	Total Delayed Recall insufficiency	1 (9)
	Recognition insufficiency	0 (0)
	**ROCF Delayed Recall** insufficiency, *n (%)*	6 (55)
	**Digit Span** insufficiency, *n (%)*	1 (9)
Attentional Functions	**Trail Making Test** Part A insufficiency, *n (%)*	2 (18)
	**Digit–Symbol-Coding (WAIS III)**	
	Satisfying	9 (82)
	Intermediate	0 (0)
	Insufficient	2 (18)
	**d2-R test** insufficiency, n (%)	
	Concentration Capacity	4 (36)
	Processing Speed	4 (36)
	Processing Accuracy (E%)	3 (27)
	**Stroop test** (Part color naming and reading) insufficiency, *n (%)*	0 (0)
Executive Functions	**Trail Making Test** Part B, *n (%)*	
	Satisfying	7 (64)
	Intermediate	1 (9)
	Insufficient	3 (27)
	**Phonemic fluency** insufficiency, *n (%)*	0 (0)
	**Semantic fluency**, *n (%)*	
	Satisfying	8 (73)
	Intermediate	3 (27)
	Insufficient	0 (0)
	**Stroop test** (Part interference), insufficiency, *n (%)*	2 (18)
	**Insufficiency > 3 tests[b]**	**4 (36)**

[a]: Insufficient/insufficiency is below the 5th percentile, and Intermediate is between the 5th and 10th percentiles of the normal population.

[b] : Excluding MOCA.

Abbreviations: AAV: Anca-associated vasculitis; FCSR: Free and Cued Selective Reminding Test; ROCF: Rey–Osterrieth Complex Figure

Supplementary Table S3: Self-Report questionnaire and cognitive trouble association

**Questionnaire on subjective cognitive complaint**	**All patients** ***n = 11***	**Cognitive impairment** ***n = 4***	**No cognitive impairment** ***n = 7***	p^a
Q3PC significant complaint[b]	6 (55)	3 (75)	3 (43)	*0.55*
Fact-Cog significant complaint[b]	4 (36)	2 (50)	2 (29)	*0.58*
MacNair significant complaint (>54) n (%)	3 (27)	2 (50)	1 (14)	*0.49*

[a]: Fisher's exact test for categorical variables.

[b]: The two subjective complaint questionnaires provided concordant results (presence or absence of cognitive complaints) for 7/11 patients (64%).

Abbreviations: Q3PC: [Questionnaire de plainte cognitive]

Supplementary Table S4: Factors associated with cognitive impairment

	**Cognitive impairment** ***n = 4***	**No cognitive impairment** ***n = 7***	p^a
Female, *n (%)*	1 (25)	4 (57)	*0.55*
Age at study enrollment, *years, median [IQR]*	75.5 [65.25 - 86.5]	69 [58.5 - 69.5]	*0.25*
Duration of disease, *months, median [IQR]*	131 [102- 149]	69 [55 - 92]	*0.16*
Present use of CS, *n (%)*	3 (75)	4 (57)	*1*
Received cumulative corticosteroid dose, *grams, median [IQR]*	28.9 [21.1 – 35.7]	18.3 [14.1-23.5]	*0.22*
Present use of IS, *n (%)*	1 (25)	2 (29)	*1*
Received induction treatment[b]:			
Cyclophosphamide	2 (50)	3 (43)	*1*
Rituximab	0 (0)	4 (57)	*0.19*
Rituximab as maintenance therapy	1 (25)	3 (43)	*1*
Sequelae of neuropathy, *n (%)*	1 (25)	5 (71)	*0.24*
VDI score, *median [IQR]*	1 [0.75 – 1.75]	2 [1.5 - 3]	*0.44*
**Brain MRI**, *n (%)*			
Hippocampal atrophy (Scheltens grade 2 or 3)	2 (50)	1 (14)	*0.49*
Fazekas 2 vascular leukoencephalopathy	1 (25)	1 (14)	*1*
Vascular sequelae (deep lacunes and junctional sequelae)	0 (0)	0 (0)	*1*
Microbleeds	0 (0)	3 (43)	*0.24*
Cortico-subcortical sequelae	0 (0)	0 (0)	*1*

[a]: Fisher's exact test for categorical variables and Mann–Whitney U test for continuous variables.

[b]: Initial diagnosis and relapse included.

Abbreviations: CS: corticosteroids, IS: immunosuppressive therapy

Supplementary Table S5: Psychopathological symptoms associated with cognitive impairment

	**All patients** ***n = 11***	**Cognitive impairment** ***n = 4***	**No cognitive impairment** ***n = 7***	p^a
**HADS-Depression (/21),** *n (%)*				*1*
No symptoms of depression (score ≤ 7)	9 (82)	3 (75)	6 (86)	
Mild symptoms of depression (score 8-10)	0 (0)	0 (0)	0 (0)	
Severe symptoms of depression (score ≥ 11)	2 (18)	1 (25)	1 (14)	
**HADS-Anxiety (/21),** *n (%)*				*0.42*
No symptoms of anxiety (score ≤ 7)	4 (36)	2 (50)	2 (29)	
Mild symptoms of anxiety (score 8-10)	3 (27)	0 (0)	3 (43)	
Severe symptoms of anxiety (score ≥ 11)	4 (36)	2 (50)	2 (29)	
**Insomnia Severity Index (/28),** *n (%)*				*0.36*
No insomnia (score 0-7)	4 (36)	1 (25)	3 (43)	
Subthreshold insomnia (score 8 -14)	4 (36)	1 (25)	3 (43)	
Moderate clinical insomnia (score 15 -21)	1 (9)	0 (0)	1 (14)	
Severe clinical insomnia (score 22 -28)	2 (18)	2 (50)	0 (0)	
**Visual Analog Scale (/10),** *n (%)*				*0.75*
No pain (score ≤ 3)	7 (64)	3 (75)	4 (57)	
Moderate pain (score 4-5)	2 (18)	0 (0)	2 (29)	
Intense pain (score ≥ 6)	2 (18)	1 (25)	1 (14)	

[a]: Fisher's exact test for categorical variables.

Abbreviations: HADS: Hospital Anxiety Depression Scale
